# Wnt5a promotes epithelial-to-mesenchymal transition and metastasis in non-small-cell lung cancer

**DOI:** 10.1042/BSR20171092

**Published:** 2017-11-29

**Authors:** Biao Wang, Zhen Tang, Huiyuan Gong, Li Zhu, Xuegang Liu

**Affiliations:** 1 Medical College of Shandong University, Jinan 250012, China; 2 Department of Thoracic Surgery, The First Affiliated Hospital, Bengbu Medical College, Bengbu 233004, China

**Keywords:** epithelial-to-mesenchymal transition, metastasis, non-small cell lung cancer, tumor models, Wnt5a

## Abstract

A recent study indicated that high Wnt5a expression is associated with poor prognosis in non-small-cell lung cancer (NSCLC) patients; however, the underlying mechanism was not clear yet. Immunohistochemistry and Western blotting were performed to examine the protein expression level in NSCLC tissues and cell lines. The role of Wnt5a in clone formation, invasiveness, migration, and epithelial-to-mesenchymal transition (EMT) of NSCLC cells was studied. Luciferase reporter assay was used to evaluate the Tcf/Lef transcriptional activity. For assessing the effects of Wnt5a on tumor growth and metastasis *in vivo*, A549 cells transfected with sh-Wnt5a were subcutaneously or orthotopically injected into nude mice. In NSCLC tissues, higher expression levels of Wnt5a and ROR2 were found, β-Catenin was expressed exceptionally, and EMT was prompted. Wnt5a overexpression increased clone formation, migration, and invasion, as well as prompted EMT of NSCLC cell *in vitro*, whereas Wnt5a knockdown showed the absolutely reversed results. Wnt5a overexpression enhanced the Tcf/Lef transcriptional activity and elevated the nuclear β-catenin level in NSCLC cells, without altering the ROR2 expression. We also demonstrated that si-β-catenin antagonized Wnt5a overexpression nduced EMT and invasiveness. Besides, *in vivo* experiment showed that sh-Wnt5a significantly increased tumor volume and tumor weight, and prompted EMT in A549 tumor-bearing mice as compared with the control. No metastasis was found in the liver tissue after sh-Wnt5a-transfected cells were orthotopically injected into nude mice as compared with the control. In conclusion, Wnt5a promotes EMT and metastasis in NSCLC, which is involved in the activation of β-catenin-dependent canonical Wnt signaling.

## Introduction

Lung cancer, which is classified into small cell lung cancer, squamous cell carcinoma (SCC), adenocarcinoma (ADC), and large cell carcinoma, remains the leading cause of cancer deaths in the world. The latter three are included in the non-small-cell lung cancer (NSCLC) classification, and ADC and SCC are known to be the most important subtypes of NSCLC [1]. NSCLC accounts for nearly 80% of lung cancer cases [2]. Though many advances have been achieved in diagnosis and treatment, the overall 5-year survival rate of lung cancer patients is only ~15% [3]. Therefore, the identification of useful biomarkers may provide potential therapeutic approaches for NSCLC.

Epithelial-to-mesenchymal transition (EMT) plays a vital role in tumor metastasis and progression in various solid tumors, particularly, in NSCLC [4,5]. EMT epithelial cells undergo morphological changes and convert into a mesenchymal cell phenotype. EMT is characterized by the loss of the epithelial adhesion molecule E-cadherin, an increase in the migratory and invasive behavior, and an increase in the mesenchymal markers vimentin and N-cadherin [6].

Wnt proteins constitute a large family of secreted lipid-modified glycoproteins, which is implicated in cellular processes such as differentiation, proliferation, apoptosis, and migration [7]. Wnt5a is known as a typical non-canonical and Wnt protein is associated with the progress and development in many malignant tumors [8]. Many previous studies have demonstrated that Wnt5a is up-regulated in various cancers, including gastric, pancreatic, and prostate cancers [6,9,10]. In contrast, it also reported to act as a tumor suppressor in cancers such as colon, thyroid, and breast [11–14]. A recent study indicated that high Wnt5a expression is associated with poor prognosis in NSCLC patients [15]. However, the underlying mechanism was not clear yet. A previous study indicated that up-regulation of Wnt5a promotes EMT and metastasis of pancreatic cancer cells [6]. We thus speculated that Wnt5a may promote NSCLC progress by promoting EMT and metastasis.

Wnt signaling can be broadly divided into two categories: the non-canonical, β-catenin-independent pathway, and the canonical, β-catenin-dependent pathway [10]. Based on the conclusion that the effects of Wnt5a on promoting EMT and metastasis of pancreatic cancer cells, cellular processes of pancreatic cancer are associated with the activation of β-catenin-dependent canonical Wnt signaling [6]. In our study, we also intend to validate which Wnt signaling pathway was involved in the development of NSCLC. The results in our study concluded that Wnt5a promotes EMT and metastasis in NSCLC, which is involved in the activation of β-catenin-dependent canonical Wnt signaling.

## Methods

### Patients and tissue samples

Formalin-fixed, paraffin-embedded NSCLC samples (ADC and SCC, *n*=20) and matched, tumor-adjacent specimens (Normal, *n*=20) were collected from patients (with an average of 63.1 years old) who underwent surgery at the First Affiliated Hospital of Bengbu Medical College, Anhui, China from 2009 to 2014. No patient received chemotherapy or radiotherapy before the operation. Informed consent has been obtained from all patients and the study protocol was approved by the Ethics Committee of the First Affiliated Hospital of Bengbu Medical College.

### Cell culture

NSCLC cell lines (A549, H1299, H1975, and H1650) and a normal human bronchial epithelial cell line (BEAS-2B) were obtained from the American Type Culture Collection (ATCC). Cells were maintained in Dulbecco’s modified Eagle’s medium (DMEM; GIBCO-BRL; Invitrogen, Carlsbad, CA, U.S.A.) supplemented with 10% FBS (Gibco, Carlsbad, CA, U.S.A.) and 1% penicillin-streptomycin (Invitrogen, Carlsbad, CA, U.S.A.) and cultured in a 5% CO_2_ incubator at 37°C.

### Cell transfection

For knockdown of endogenous Wnt5a expression in NSCLC cells, siRNA technology was used. The target sequence was 5′-GTTTTGGCCACTGACTGA-3′. The Wnt5a siRNA expression cassette was subcloned into the expression vector pcDNA 6.2. Wnt5a-expressing plasmid was constructed by subcloning the human Wnt5a cDNA into the pcDNA3.1vector (Invitrogen, Carlsbad, CA, U.S.A.). si-Wnt5a was transfected into A549 cells and Wnt5a-expressing plasmid was transfected into H1975 cells. A549 cells that transfected with the empty expression vector pcDNA6.2 and H1975 cells that transfected with the empty expression vector pcDNA3.1 were the corresponding controls. Cell transfection was performed using the Lipofectamine 2000 Transfection Reagent, according to the manufacturer’s instructions (Invitrogen, Carlsbad, CA, U.S.A.).

si-β-Catenin was purchased from GenePharma company (Shanghai, China). For assessment of the role of β-catenin, NSCLC cell line H1975 was transfected with si-β-catenin (20 nM) for 24 h. The transfection efficiency was determined by the Western blot analysis. After that, cells were tested for invasion and migration ability.

### Luciferase reporter assay

Cells were co-transfected with pcDNA 6.2 (Control), pcDNA 6.2-si-Wnt5a, pcDNA3.1 (Control), pcDNA3.1- Wnt5a, and 300 ng TopFlash/FopFlash plasmid (Millipore). Cells were harvested 36 h after transfection and lysed in 100 µl of passive lysis buffer (Promega), and 25 µl lysates were analyzed for luminescent signal. The reporter and *Renilla* luciferase activities were monitored using Dual Luciferase reporter system (Promega).

### Immunohistochemistry

Examination of the expression and distribution of Wnt5a, β-catenin, and ROR2 in NSCLC tissues was performed by the immunohistochemical method. Briefly, 4-μm paraffin-embedded sections were deparaffinized and rehydrated. For the blockage of endogenous peroxidase activity, 3% hydrogen peroxide was used. After antigen retrieval, sections were incubated with the primary antibodies against Wnt5a, β-catenin, and ROR2 (each diluted in 1:50) at 4°C overnight. Biotinylated secondary antibodies were then used to treat the tissues sections, followed by incubation with streptavidin–horseradish peroxidase complex (Santa Cruz Biotechnology Inc., Santa Cruz, California, U.S.A.). Immunoreactivity was visualized with diaminobenzidine (Sigma–Aldrich, St. Louis, MO, U.S.A.). The sections were counterstained with Hematoxylin. For blank controls, the primary antibody was omitted.

### Clongenic assay

Cells were seeded in six-well plates (10^3^ cells/well) for 6 h. Then the medium was discarded, and fresh medium was added to the wells, after which cells were allowed to grow for 14 days to form colonies, which were stained with Crystal Violet (0.4 g/l; Sigma). The images were collected, and the number of colonies in each well was counted.

### Cell migration and invasion assays

Assays were performed using a standard Boyden chamber protocol (Costar; Corning Inc., Lowell, MA, U.S.A.). In brief, the cells (5 × 10^4^ per well) were detached using enzyme-free cell dissociation solution and suspended in 500 µl RPMI-1640 medium. Cells in 0.2 ml of medium were seeded on a transwell apparatus and 600 µl of medium containing 20% FBS was added to the lower chamber. The invasion assay was performed following the same procedure; however, the filters of the transwell chambers were coated with 30 µg Matrigel (BD Biosciences, San Jose, CA, U.S.A.). Cells were allowed to migrate toward the complete medium for 12 h in the migration assay or 24 h in the invasion assay. Non-migrating cells were removed with a cotton swab and by PBS washes. The Crystal Violet assay was used to quantitate the number of migrating or invading cells. Values for invasion and migration were obtained by counting five fields per membrane under a microscope (×200) and represent the average of three independent experiments.

### Western blot analysis

Protein was collected from tissues, and the cytoplasmic and nuclear proteins were separately isolated using the Proteo JET Cytoplasmic and Nuclear Protein Extraction Kit, according to the manufacturer’s instructions (Fermentas, Burlington, ON, Canada). Protein was lysed in radioimmunoprecipitation buffer (RIPA) containing protease inhibitors at 4°C for 30 min. Lysates were prepared with a RIPA lysis buffer kit (Santa Cruz Biotechnology, Inc.), and the protein concentrations were quantitated using a Bio–Rad protein assay (Bio–Rad Laboratories, Inc., Hercules, CA, U.S.A.). Proteins (30 μg) were resolved by SDS/PAGE, transferred on to nitrocellulose membranes, and probed with the primary antibodies to the detected proteins mentioned above, and then horseradish peroxidase conjugated secondary antibodies, respectively. Anti-β-actin antibody was used as a loading control. Detection was done using an ECL system (GE Healthcare Life Sciences, Piscataway, NJ, U.S.A.).

### shRNA transfection

Wnt5a shRNA plasmid and control shRNA plasmid were provided by Takara (Dalian, China). A549 cells were seeded in six-well plates at 2.0 × 10^4^ cells/well, and cultured overnight to 80% confluence prior to transfection. Transfection was performed using Lipofectamine Plus (Grand Island, NY, U.S.A.), and the ratio of the plasmids and the transfection reagent was 1 mg:2 ml. Cells were transfected with plasmid as per the manufacturer’s instructions.

### Orthotopic tumor model

Female BALB*/c nu/nu* mice (age range, 6 weeks) were purchased from Tumor Research Institute, Chinese Academy of Sciences (Beijing, China). Mice were anesthetized by peritoneal injection of chloral hydrate at 0.4 mg/g body weight. A 5-mm skin incision overlying the left chest wall was made and the left lung was visualized through the pleura. A549 cells (3 × 10^6^ ) that transfected with sh-Wnt5a or sh-control in 50 μg of growth factor reduced Matrigel (BD Biosciences) in 50 μl of Hank’s balanced salt solution were injected into the left lungs of the mice through the pleura using a 30-gauge needle. After tumor cell injection, the wound was stapled and the mice were placed in the left lateral decubitus position and observed until fully recovered. To detect the effects of Wnt5a on NSCLC invasion and metastasis *in vivo*, nude mice were anesthetized and killed, and liver tissues were resected, fixed in formalin, embedded in paraffin, and cut into sections. The sections were stained with H&E, as described above.

### Subcutaneous tumor model

Female BALB*/c nu/nu* mice (age range, 6 weeks) were purchased from Tumor Research Institute, Chinese Academy of Sciences (Beijing, China). Single-cell suspensions containing 3 × 10^6^ A549 cells that transfected with sh-Wnt5a or sh-control in 0.1 ml of Hank’s balanced salt solution were injected subcutaneously into rear right flank of each BALB*/c nu/nu* mouse. After 10–15 days, the tumor models were established. Tumor volumes were measured every 3 days. On the 30th day, mice in all groups were killed and tumors were weighed. Results were plotted as relative tumor weight and volume for the first day of the treatment up to the final day. The relative tumor weight is relative to that of the control (vehicle) designated as 100.

### Statistical analysis

Data were shown as mean ± S.D. By using the Student’s *t*test or one-way ANOVA followed by the Tukey’s test, differences in the means were determined. A *P*-value of <0.05 was considered statistically significant.

## Results

### The expression of Wnt5a, ROR2, β-Catenin, and EMT-related proteins in NSCLC tissues and cells

**Figure 1:**
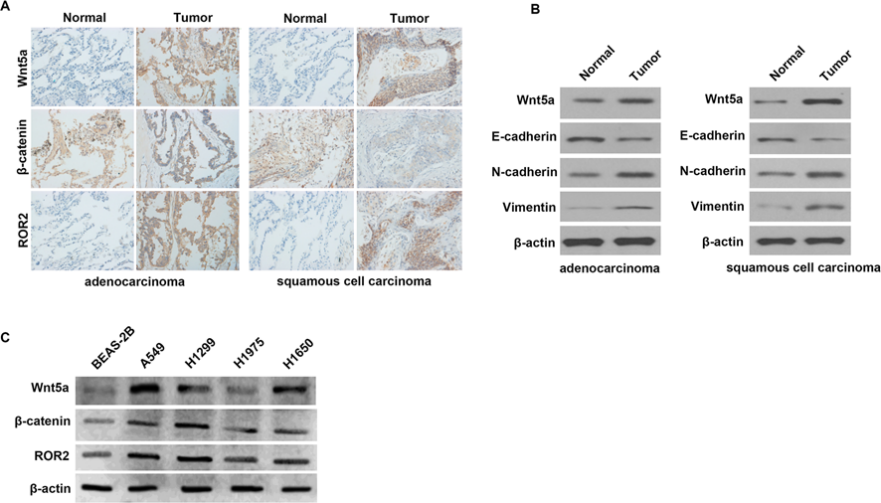
The expression of Wnt5a, ROR2, β-Catenin, and EMT-related proteins in NSCLC tissues and cells (**A**) The expression levels of Wnt5a, ROR2, and β-Catenin in NSCLC (ADC and SCC) and matched adjacent normal lung tissues by using the immunohistochemistry analysis. (**B**) The expression of Wnt5a and EMT-related proteins E-cadherin, N-cadherin, and vimentin in NSCLC tissues and matched adjacent normal lung tissues by using the Western blotting. (**C**) The expression levels of Wnt5a, ROR2, and β-Catenin in NSCLC and normal cell lines by using the Western blotting.

We examined Wnt5a, ROR2, and β-Catenin expression in NSCLC tissues (ADC and SCC) and matched adjacent normal lung tissues by the immunohistochemistry analysis. The results (Figure 1A) indicated that higher expression of Wnt5a and ROR2 were found in ADC and SCC tissues than that in the matched, adjacent non-tumor tissues. Besides, we found that β-Catenin was expressed exceptionally in ADC and SCC tissues.

We also detected the expression of Wnt5a and EMT-related proteins E-cadherin, N-cadherin, and vimentin in NSCLC tissues; the results (Figure 1B) showed that the expression of Wnt5a was higher in NSCLC tissues than that in adjacent normal lung tissues, which is consistent with the immunohistochemistry analysis. The expression of E-cadherin was lower in ADC and SCC tissues than that in the adjacent normal lung tissues, but the expression of N-cadherin and vimentin were higher in ADC and SCC tissues than that in the adjacent normal lung tissues.

To confirm the results of immunohistochemistry, the Western blotting (Figure 1C) was used to examine Wnt5a, ROR2, and β-Catenin expression in NSCLC cell lines. We found that the expression levels of Wnt5a, ROR2, and β-Catenin were higher in NSCLC cell lines A549, H1299, H1975, and H1650 than that in normal cell line BEAS-2B. Particularly, their expression levels were highest in A549 cells and were lowest in H1975 cells.

### Wnt5a increases clone formation, migration, and invasiveness of NSCLC cell *in vitro*

**Figure 2:**
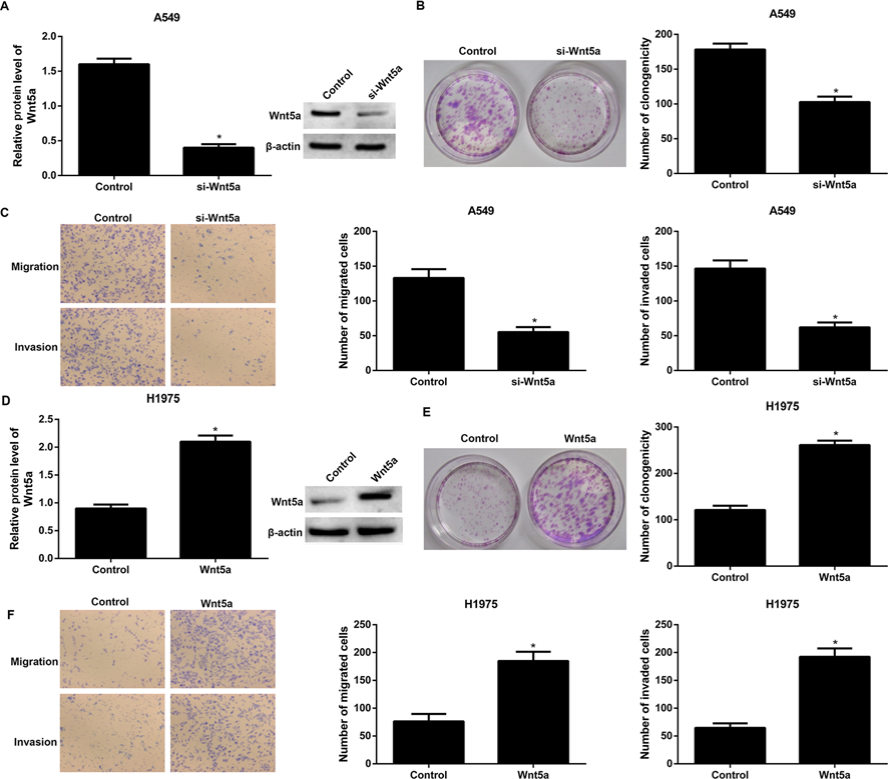
Wnt5a increases clone formation, migration, and invasiveness of NSCLC cell *in vitro* (**A**) Wnt5a was knocked down in A549 cells, which was determined by the Western blotting. (**B**) siRNA-mediated silencing of Wnt5a profoundly reduced clone formation of A549 cells. (**C**) siRNA-mediated silencing of Wnt5a profoundly reduced migration and invasiveness of A549 cells. (**D**) Wnt5a was overexpressed in H1975 cells, which was determined by the Western blotting. (**E**) Overexpression of Wnt5a profoundly increased clone formation of A549 cells. (**F**) Overexpression of Wnt5a profoundly increased migration and invasiveness of A549 cells. A549 cells that transfected with the empty expression vector pcDNA6.2 and H1975 cells that transfected with the empty expression vector pcDNA3.1 were the corresponding controls. *P<0.05 compared with the control group.

To examine the biological functions of Wnt5a in NSCLC, Wnt5a was knocked down in A549 cells or overexpressed in H1975 cells, which were determined by the Western blotting, respectively (Figure 2A,D). Our results showed that siRNA-mediated silencing of Wnt5a profoundly reduced clone formation, migration, and invasiveness of A549 cells (Figure 2B,C) in comparison with the cells transfected with the empty vector. Moreover, the clone formation, migration, and invasiveness were significantly increased in Wnt5a-overexpressing H1975 cells than in empty vector transfected cells (Figure 2E,F).

### Wnt5a induces EMT in NSCLC cells

**Figure 3:**
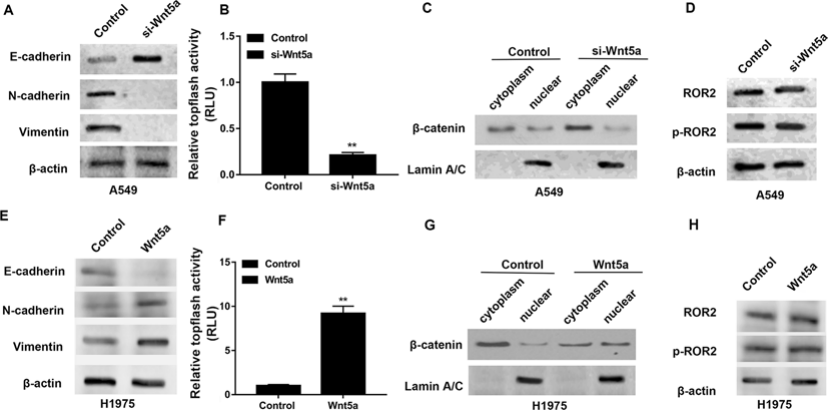
Wnt5a induces EMT in NSCLC cells (**A**) The effects of siRNA-mediated silencing of Wnt5a on the expression of N-cadherin, vimentin, and E-cadherin in A549 cells by the Western blotting analysis. (**B**) Luciferase reporter assays using TopFlash/FopFlash reporter plasmids to monitor the activity of Tcf/Lef transcription factor. (**C**) The effects of Wnt5a knockdown on the expression level of nuclear β-catenin. Lamin A/C was used as a nuclear internal control by the Western blotting analysis. (**D**) The effects of Wnt5a knockdown on the expression levels of ROR2 and p-ROR2 in A549 cells by the Western blotting analysis. (**E**) The effects of Wnt5a overexpression on the expression of N-cadherin, vimentin, and E-cadherin in A549 cells by the Western blotting analysis. (**F**) Luciferase reporter assays using TopFlash/FopFlash reporter plasmids to monitor the activity of Tcf/Lef transcription factor. (**G**) The effects of Wnt5a overexpression on the expression level of nuclear β-catenin. Lamin A/C was used as a nuclear internal control by the Western blotting analysis. (**H**) The effects of Wnt5a overexpression on the expression levels of ROR2 and p-ROR2 in A549 cells by the Western blotting analysis. A549 cells that transfected with the empty expression vector pcDNA6.2 and H1975 cells that transfected with the empty expression vector pcDNA3.1 were the corresponding controls.

We next checked the effect of Wnt5a on the EMT of NSCLC cells. The Western blotting analysis demonstrated that siRNA-mediated silencing of Wnt5a inhibited EMT by decreasing the expression of N-cadherin and vimentin, as well as by increasing the expression of E-cadherin in A549 cells (Figure 3A). We evaluated the activity of Tcf/Lef transcription factor, involved in the Wnt signaling pathway, by luciferase reporter assays. As shown in Figure 3B, knockdown of Wnt5a decreased Tcf/Lef transcriptional activity in A549 cells. Then, the cytoplasm and nucleus were separated and used for the detection of β-Catenin. Western blot analysis revealed that siRNA-mediated silencing of Wnt5a raised the cytoplasm level of β-catenin without altering the total level of the protein in A549 cells, indicating that β-catenin is prevented from entering the nucleus (Figure 3C). The nuclear lamina is a filamentous structure subtending the nuclear envelope and required for chromatin organization, transcriptional regulation, and maintaining nuclear structure [16]; thus, Lamin A/C was used as a nuclear internal control. We also found that Wnt5a knockdown had no effects on the expression levels of ROR2 and p-ROR2 both in A549 cells that transfected with si-Wnt5a and empty vector (Figure 3D).

Moreover, we found that Wnt5a-overexpressing NSCLC cells undergo an EMT, as evidenced by an increase in the expression of N-cadherin and vimentin and a concomitant reduction in the E-cadherin expression in H1975 cells (Figure 3E). As shown in Figure 3F, overexpression of Wnt5a enhanced Tcf/Lef transcriptional activity in H1975 cells. Western blot analysis revealed that Wnt5a overexpression raised the nuclear level of β-catenin without altering the total level of the protein in H1975 cells, indicating a translocation of β-catenin from the cytoplasm to the nucleus (Figure 3G). We also found that Wnt5a overexpression had no effects on the expression levels of ROR2 and p-ROR2 both in H1975 cells that transfected with Wnt5a overexpression and empty vectors (Figure 3H).

### si-β-catenin reversed the effects of Wnt5a overexpression on H1975 cells

**Figure 4:**
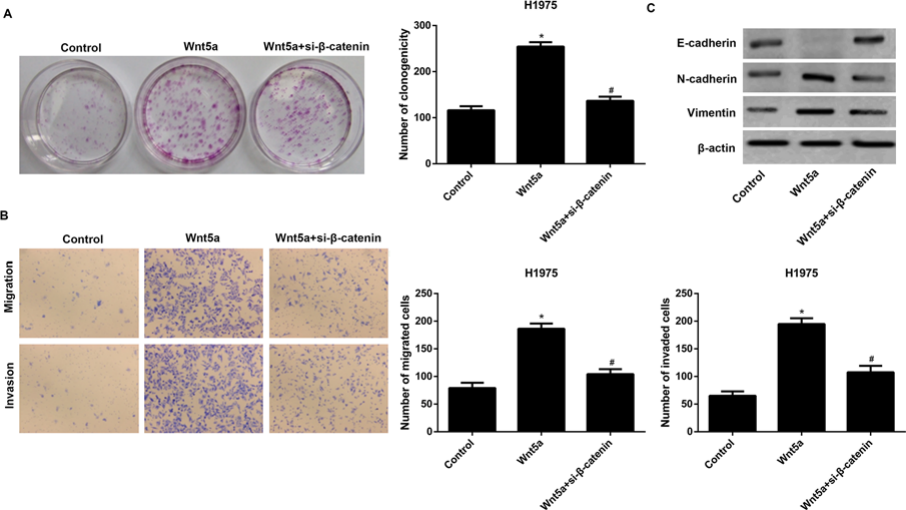
si-β-catenin reversed the effects of Wnt5a overexpression on H1975 cells (**A**) si-β-catenin reversed the effects of Wnt5a overexpression on clone formation in H1975 cells. (**B**) si-β-catenin reversed the effects of Wnt5a overexpression on cell migration and invasion. (**C**) si-β-catenin reversed the effects of Wnt5a overexpression on EMT in H1975 cells. H1975 cells that transfected with the empty expression vector pcDNA3.1 were the control. *P<0.05 compared with the control group and ^#^*P*<0.05 compared with the Wnt5a overexpression group.

Notably, si-β-catenin reversed the promotion of clone formation by Wnt5a overexpression in H1975 cells (Figure 4A). Moreover, si-β-catenin reversed the promotion of cell migration and invasion by Wnt5a overexpression (Figure 4B). si-β-catenin also blocked Wnt5a overexpression nduced EMT in H1975 cells (Figure 4C).

### The effects of Wnt5a knockdown on tumor volume, tumor weight, EMT-related proteins, and metastasis *in vivo*

**Figure 5:**
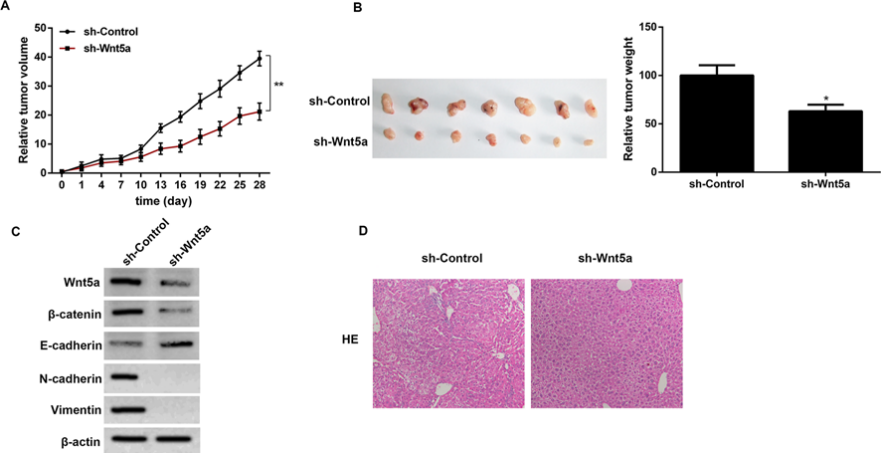
The effects of Wnt5a knockdown on tumor volume, tumor weight, EMT-related proteins, and metastasis *in vivo* (**A**) The effect of Wnt5a knockdown on tumor volume at 0–28 days in A549 tumor-bearing mice. (**B**) The effect of Wnt5a knockdown on tumor weight at 28th day in A549 tumor-bearing mice. (**C**) The effect of Wnt5a knockdown on the expression levels of β-catenin, E-cadherin, N-cadherin, and vimentin in A549 tumor-bearing mice. (**D**) The effect of Wnt5a knockdown on NSCLC invasion and metastasis *in vivo* by pathological examination. *P<0.05 compared with the control group and * *P<0.01 compared with the control group.

The animal experimental results showed that sh-Wnt5a significantly increased tumor volume and tumor weight in A549 tumor-bearing mice as compared with the control (Figure 5A,B). Besides, we detected the expression levels of β-catenin, E-cadherin, N-cadherin, and vimentin in NSCLC tumor. The results showed that the expression levels of β-catenin, N-cadherin, and vimentin were decreased in sh-Wnt5a mice as compared with that in the sh-control group (Figure 5C). We further assessed the effects of Wnt5a on NSCLC invasion and metastasis *in vivo.* A549 cells transfected with sh-Wnt5a were orthotopically injected into nude mice. The results showed that no metastasis was found in the liver tissue, as determined by pathological examination. Regarding the empty vector transfected cells, metastatic tumors were formed when they were inoculated into nude mice (Figure 5D).

## Discussion

Wnt5a was found to be up-regulated in solid tumors, like pancreatic cancer, skin cancer, and gastric cancer [6,17]. Our results confirm the previous finding that there is an elevation in Wnt5a expression in NSCLC tissues compared with adjacent normal lung tissues [18]. We also found the elevation in Wnt5a expression in NSCLC cell lines compared with normal cell lines. Moreover, we found that Wnt5a overexpression increased clone formation, migration, and invasiveness of NSCLC cell *in vitro*, whereas Wnt5a knockdown showed the absolutely reversed results. Other studies indicated that transfection of Wnt-5a into NSCLC cell lines stimulated cell proliferation, whereas Wnt-5a siRNA suppressed proliferation [19]. Wnt5a may also protect cells from apoptosis by activation of protein kinase C and Akt and may decrease cellular adhesion by reducing cadherin expression [20,21].

Results also indicated that, when compared with adjacent normal tissues, NSCLC tissues had elevated expression of vimentin and N-cadherin and reduced expression of E-cadherin, indicating the presence of EMT. In Wnt5a-overexpressing NSCLC cells, elevated expression of vimentin and N-cadherin and reduced expression of E-cadherin were also found. However, in Wnt5a-knockdown NSCLC cells, these protein expression levels were reversed. *In vitro* studies further confirmed the inhibition of EMT in sh-Wnt5a mice, as evidenced by decreased expression of vimentin and N-cadherin and increased expression of E-cadherin. Our study is consistent with the previous studies [6,10,22]. For example, Kanzawa et al. [10] suggested that Wnt5a regulates the induction of EMT and the maintenance of cancer stem cell properties in MKN-7 cells. Wnt5a may play an important role in constructing an advantageous tumor microenvironment for the progression and development of human gastric carcinoma.

Metastasis remains a major cause of morbidity and mortality in cancer patients. Wnt5a is reported to regulate numerous biological events associated with metastasis. Yamamoto et al. [23] reported that Wnt5a contributes to gastric cancer cell dissemination to the liver through up-regulation of laminin γ 2. In agreement with the report by Ripka et al. [24], our *in vitro* experiment demonstrated that Wnt5a acts as a potent activator of NSCLC cell migration and invasion. Our *in vivo* study showed that no metastasis was found in the liver tissue after sh-Wnt5a-transfected cells were orthotopically injected into nude mice as compared with the control. Regarding the empty vector transfected cells, metastatic tumors were formed when they were inoculated into nude mice. Using an orthotopic pancreatic cancer model, researchers demonstrated that Wnt5a-overexpressing cancer cells formed metastatic tumors at multiple sites, whereas control cells failed to metastasize [6].

Another study indicated that overexpression of ROR2 and Wnt5a co-operatively correlates with unfavorable prognosis in patients with NSCLC [15]. In our study, overexpression of ROR2 was observed in NSCLC tissues and cell line. Wnt5a can exert its biological effects through the canonical or non-canonical Wnt signaling pathway, largely depending on the availability of specific receptors [25]. We showed that the protein level of ROR2 receptor remained unchanged after Wnt5a knockdown or overexpression. Accumulating evidence indicates an important role of β-catenin signaling in the pathogenesis of NSCLC [26,27]. It is well known that the activation of the β-catenin signaling is related to the initiation of EMT [28]. In agreement with these findings, the luciferase reporter assays showed that knockdown of Wnt5a decreased Tcf/Lef transcriptional activity, whereas overexpression of Wnt5a enhanced Tcf/Lef transcriptional activity. Moreover, our *in vitro* data demonstrated that si-β-catenin antagonized Wnt5a overexpression nduced EMT and invasiveness, indicating that β-catenin signaling may involve in the effects of Wnt5a on NSCLC progress. To sum up, this study confirms an up-regulation of Wnt5a in NSCLC tissues and cells. Wnt5a plays an important role in regulating NSCLC clone formation, cell migration, and invasion *in vitro*. Wnt5 also exerts effects on tumor growth, EMT, and metastasis *in vivo*. We further confirmed that Wnt5a contributes to NSCLC progress through activation of β-catenin-dependent canonical Wnt signaling.

## Abbreviations

ADC: adenocarcinoma
EMT: epithelial-to-mesenchymal transition
H&E: Hematoxylin and Eeosin
NSCLC: non-small-cell lung cancer
RIPA: radioimmunoprecipitation buffer
ROR2: receptor tyrosine kinase like orphan receptor 2
SCC: squamous cell carcinoma
Wnt5a: Wnt family member 5A
